# The role of histone lysine demethylases in cancer cells’ resistance to tyrosine kinase inhibitors

**DOI:** 10.20517/cdr.2019.16

**Published:** 2019-06-19

**Authors:** Jasmine Cassar White, Perla Pucci, Francesco Crea

**Affiliations:** School of Life Health and Chemical Sciences, The Open University, Walton Hall, Milton Keynes MK76AA, UK.

**Keywords:** Epigenetics, drug resistance, cancer

## Abstract

Current cancer therapies are often associated with treatment failure and reduced patients’ survival due to drug resistance. There are various mechanisms involved in the acquisition of cancer drug resistance, including the selection of advantageous mutations, overexpression of transporter proteins and epigenetic alterations. In this context, epigenetic alterations refer to chromatin-mediated regulation of gene expression that results in heritable changes in the cellular phenotype. There is an ever-growing body of evidence suggesting that epigenetic mechanisms play an important role in bringing about drug resistance in cancer cells. While the relationship between chemotherapy and epigenetics has been widely discussed, emerging evidence indicates that specific epigenetic effectors are also crucial for the development of resistance to tyrosine kinase inhibitors (TKIs). One particular gene that encodes the histone lysine demethylase KDM5A is overexpressed in several cancers. In breast cancer tissues, cells with *KDM5A* gene amplification were found to be more resistant to erlotinib, an inhibitor of the tyrosine kinase epidermal growth factor receptor (EGFR), when compared to cells without the same amplification. KDM5A was also shown to mediate resistance to a second EGFR inhibitor called gefitinib, in EGFR-mutant lung cancer cell lines. This evidence indicates that KDM5A could activate alternative survival pathways involved in overcoming EGFR inhibition. In line with these results, another histone demethylase (i.e., KDM1A) promotes liver cancer cells’ resistance to the TKI sorafenib. Current evidence provides a suitable rationale to consider the use of specific KDMs inhibitors to sensitize cells to tyrosine kinase targeted therapies and thus, presents an opportunity to prevent the further development of drug resistance. This review discusses the involvement of histone lysine demethylases in the development of resistance to TKI and highlights the importance to develop new cancer treatment regimens to counteract this phenomenon.

## Introduction

The introduction of targeted therapies against cancer-specific molecules and signaling pathways led to significant improvements in the quality of medical care for cancer, wider therapeutic indices and more limited non-specific toxicities, when compared to earlier forms of cancer therapies^[1]^. Tyrosine kinases (TKs) are particularly important targets because they play a key role in the modulation of growth factor signaling, therefore influencing many downstream pathways^[2]^. In recent years, numerous tyrosine kinase inhibitors (TKIs) have been developed as highly effective anti-tumor and anti-leukemic agents^[3,4]^. Unfortunately, intrinsic TKI resistance^[5-7]^ and acquired therapeutic resistance^[8-10]^ to TKIs often develops along the course of therapy, reducing TKIs clinical efficacy and hampering effective treatment of cancer.

There are many molecular mechanisms that are involved in the acquisition of resistance to TKIs, such as mutation of drug targets, changes in drug metabolism and the over-expression of cancer drug resistance transporter proteins that result in increased rates of drug efflux^[11]^. Apart from this, tumors are highly adaptable to the biological microenvironment and changes in the activation and inactivation patterns of survival signaling pathways can also result in the emergence of drug resistance^[12,13]^. Epigenetic mechanisms have been found to play an important role in generating drug resistance in cancer cells^[14,15]^. In this context, epigenetic alterations refer to chromatin-mediated regulation of gene expression that results in heritable changes in the cellular phenotype^[16]^.

In this review we will focus on the role of a specific family of epigenetic effectors (i.e., histone lysine demethylases, KDMs) in the context of TKIs resistance.

## Tyrosine kinases and inhibitors: hysiological functions and relevance in cancer

TKs are enzymes capable of selectively phosphorylating tyrosine residues in different substrates, resulting in the activation of numerous proteins involved in the signal transduction cascade^[17]^. Therefore TKs play key roles in mediating biological processes such as cellular differentiation, metabolism, growth and apoptosis in response to both external and internal stimuli^[18]^. For example, FMS Like Tyrosine Kinase 3 (FLT3) is a class III TK cytokine receptor that is expressed on the surface of immature hematopoietic progenitor cells and plays important roles in promoting the survival and correct growth of progenitor cells and hence, the control of hematopoiesis^[19]^. *FLT3* mutations can be found in patients suffering from acute myeloid leukaemias (AMLs) and B-cell acute lymphoblastic leukaemias (ALLs) and cause uncontrolled receptor activation, constitutive FLT3 signalling and as a result, activation of the STAT4, RAS/MAPK and PI3K pathways important for cell division, apoptosis and cell formation^[20]^.

Due to their wide roles in many signaling pathways, the level of intra-cellular tyrosine kinase phosphorylation must be tightly controlled; this is achieved through maintenance of the balance between TKs and their antagonists, tyrosine phosphatases.

Despite being strictly regulated in physiological conditions, TKs may acquire aberrant functions caused by various mechanisms including mutations and overexpression of the *TK* genes, leading to constitutive oncogenic TKs activation and development of malignant phenotypes^[21]^. There are four main mechanisms resulting in the constitutive activation of receptor TKs in human cancers: (1) gain-of-function mutations; (2) overexpression and genomic amplifications; (3) chromosomal rearrangements; and/or (4) autocrine activation^[17]^.

Gain-of-function mutations in TKs lead to abnormal downstream signal transduction and can be exemplified by “driver mutations” that result in a selective growth advantage to cells. This is an important aspect of cancer initiation and progression^[22]^. Overexpression of TKs, as a result of gene amplification, in human cancers leads to an increase in the local concentration of the receptor TKs and consequent elevation of TK signaling^[23]^. Chromosomal rearrangements lead to the formation of new TK fusion oncoproteins, such as the BCR-ABL fusion protein^[24]^. Identification of such chromosomal rearrangements and the associated fusion oncoprotein can be instrumental to the development of new therapeutics as these fusion proteins are often good targets for small molecule inhibitors. Finally, autocrine activation refers to a situation where the cells are constantly secreting extra-cellular ligands that bind to receptors on the same cells, leading to the activation of specific TK pathways^[25]^. Autocrine activation of TKs has been identified and studied in various types of cancers, including HGF-MET in AML^[26]^ and SCF-KIT autocrine loop in small cell lung cancer^[27]^.

The effect of constitutively active TKs can be blocked through the use of TKIs, which have been found to be effective in the targeted treatment of various malignancies^[1]^. Imatinib was the first TKI to be developed for use against chronic myelogenous leukemia (CML)^[14]^ and was the first TKI successfully introduced in clinical oncology^[28]^. Imatinib targets the BCR-ABL TK that is selectively expressed by CML cells and promotes their uncontrolled proliferation.

Since then, numerous TKIs have been discovered and developed as anti-cancer treatments targeting a vast array of cancer types. These inhibitors include gefitinib^[29,30]^ and erlotinib; two oral anti-cancer treatments that act as selective inhibitors of the TK domain of the EGFR. These drugs are approved for the treatment of lung cancer^[22,23]^ and non-small cell lung cancer and pancreatic cancer^[31-33]^ respectively. Ibrutinib is a first-in-class small molecule inhibitor of Bruton’s tyrosine kinase (BTK) and is used to treat B cell cancers such as mantle cell lymphoma^[34]^ and Waldenström’s macroglobulinemia^[35]^.

Subsequently, the activity of TKIs has been widened by designing molecules that target more than one enzyme. For example Sunitinib, an oral, small-molecule, multi-targeted receptor (PDGFR and VEGFR families) TKI was the first cancer drug to be approved for two indications, renal cell carcinoma (RCC) and imatinib-resistant gastrointestinal stromal tumour^[36]^, at the same time. Most recently, dasatinib, another multi-targeted TKI developed to contrast imatinib resistance^[37]^ and enhance TKI tolerability^[38]^ in patients with CP-CML, was approved for the treatment of CML and ALL^[39,40]^.

Cabozantinib is a newly developed small molecule inhibitor of the tyrosine kinases c-MET and VEGFR2 that is used to treat medullary thyroid cancer^[41]^ and a first-line treatment for advanced RCC^[42]^, amongst other cancer types. Vandetanib is a small molecule TKI that targets key signaling pathways in cancer by inhibiting VEGFR-dependent tumour angiogenesis and EGFR- and RET-dependent cell proliferation and survival, and is used to treat tumours of the thyroid gland^[43,44]^. Trametinib is another first-in-class TKI, that acts as an allosteric inhibitor of MEK1 and MEK2 and which is approved for treatment of metastatic melanoma harboring the BRAF V600 mutation^[45]^.

Interestingly, the TKI ruxolitinib was the first small molecule inhibitor of JAK1/2 kinases which was used in the treatment of myelofibrosis, applied to the field of myeloproliferative neoplasms^[46]^ and as a result of the promising results achieved in clinical trials, approved for the treatment of myelofibrosis by the U.S. FDA^[47,48]^. The most recent receptor tyrosine kinase to be approved is lorlatinib, the first third-generation anaplastic lymphoma kinase (ALK) inhibitor approved for the treatment of patients with ALK-positive metastatic non-small cell lung cancer^[49]^.

It is important to note that while the small molecules mentioned above, are mainly used to target TKs, other types of inhibitors such as monoclonal antibodies (mAbs) can also be used to target TKs. At present, more than 70 mAbs have been approved by the EMEA and FDA for therapeutic use^[50]^ and the number of approvals is rapidly increasing. The first two tyrosine kinases to be targeted by mAbs were the growth factor receptors EGFR and HER2. However, these treatments are currently being challenged by recently emerging therapeutics as a result of their associated side effects and the development of resistance.

## KDMs and TKI resistance

While the relationship between chemotherapy and epigenetics has been widely discussed^[51,52]^, emerging evidence indicates that specific epigenetic effectors are also crucial for the development of resistance to TKIs in the treatment of cancer.

Histones post-translational modifications (HPTMs) provide a mechanism for the regulation of gene expression that is transmissible from parent to offspring. The globular domain and unstructured C- or N-terminal tails of histones are subject to various covalent modifications including acetylation, phosphorylation and methylation as well as the additions of large groups such as ubiquitin and ADP-ribose^[53]^.

These HPTMs contribute to the control of gene expression in a context-dependent manner, by influencing the compaction of chromatin or through signaling and recruitment of other protein complexes^[53]^. An appropriate balance between the stability and dynamics of HPTMs is required for accurate gene expression. Carcinogenesis and tumorigenesis are highly dependent on the dysregulation of normal gene expression and thus, HPTMs such as methylation and demethylation, play a critical role in tumour progression^[54]^. As a result, enzymes that catalyse the PTMs (e.g., histone lysine methylases) and their removal (e.g., histone lysine demethylases) are actively being pursued as small-molecule targets for the development of new oncology therapeutics.

KDMs are a group of enzymes that catalyze the removal of mono- (me1), di- (me2) and tri-methyl (me3) marks on histones lysine residues^[55]^. In particular, genes encoding for various KDMs have been found to be overexpressed in several cancers^[56-58]^. In addition to this, some KDMs have been found to confer resistance to established TKIs [Table 1].

**Table 1:** KDMs and their links to human cancers tyrosine kinase inhibitor treatment resistance

Enzyme	Cancer	TKI	Ref.	KDM inhibitors in clinical trials
KDM1A	Non-small cell lung cancer	Gefitinib	[67]	4SC-202^[68]^ ORY-1001^[69]^ Tranylcypromine^[70,71]^
	Hepatocellular carcinoma	Sorafenib	[72]	
KDM5A	Breast cancer	Erlotinib	[73]	
	Lung cancer	Gefitinib	[74]	
KDM5B	Non-small cell lung cancer	Gefitinib	[67]	
KDM5C	Lewis lung carcinoma, renal cell carcinoma	Sunitinib	[75]	
KDM6A	TEL-ABL-positive acute lymphoblastic leukaemia	Imatinib	[76]	

TKI: tyrosine kinase inhibitor

Over 20 KDMs enzymes have been identified thus far.

KDMs can be classified into two broad categories, depending on their catalytic mechanism of action and sequence homology: (1) lysine-specific demethylases (LSDs or KDM1 family); (2) Jumonji C-containing histone demethylases (JmjC KDMs or KDM2-8 families)^[59]^.

Both categories of KDMs use oxidative mechanisms to catalyze N-methyl-lysine demethylation albeit in somewhat different manners. LSDs employ Flavin adenine dinucleotide and electron transfer in their mechanism of action. As a result of this, LSDs are unable to demethylate tri-methylated lysine residues on histones since the required electron lone pair is only present on mono- and di-methylated histones^[59]^. The second family of JmjC KDMs uses 2-oxoglutarate and O_2_ as co-substrate, with Fe(II) employed as a cofactor for the enzymatic oxygenase reaction^[59]^. This means that JmjC demethylases can remove mono-, di- and tri-methyl marks on lysine residues of histones^[59]^.

The exact biological functions of KDMs are poorly understood. Having said this, there is significant evidence to suggest that many of these enzymes play an important role in the early stages of growth, development and differentiation of embryonic stem cells^[60,61]^. This is evidenced by pre-clinical experiments demonstrating that knockdown of KDM8 in mice embryos leads to protein 53 (p53) upregulation and thus, resulting in active resorption at early stages of development^[62]^. Strobl-Mazzulla *et al.*^[63]^ also report that KDM4A is required for the expression of neural crest specifier genes in embryonic chicken since knockdown of KDM4A in these cells leads to a significant decrease in the expression of the said genes.

Apart from being involved in the early stages of embryonic development and differentiation, KDMs have been shown to exhibit dysregulated expression patterns in many cancer types^[60]^. This can have a myriad of effects on the cell functions, including transcriptional activation of tumour oncogenes, transcriptional repression of tumour suppressors, disruption of chromosomal stability as well as interaction with key hormonal receptors that control cellular proliferation^[64-66]^. The role of KDMs in mediating these pathways can also have implications in cancer TKI resistance. Table 1 summarizes the alterations of various KDMs and their links to TKI cancer treatment resistance.

Recently, some KDMs such as KDM1A and KDM5A, have been found to be critical epigenetic factors for the development of resistance to TKIs such as erlotinib^[73]^, sorafenib^[72]^ and gefitinib^[67,74]^. The relationship between KDMs and this resistance in various tumours is emerging from recent studies and seems to be a promising field to pave the way for future potential clinical applications^[67,72-76]^. Hence, there is a growing body of evidence documenting this interaction and the various roles played by KDMs in mediating TKI resistance^[67,72-76]^.

Hou *et al.*^[73]^ investigated the role played by KDM5A in breast cancer. The authors reported that breast cancer cells with *KDM5A* gene amplification and hence, with up-regulated KDM5A mRNA and protein levels, were found to be more tolerant to the EGF receptor TKI erlotinib when compared to cells without the same amplification^[62]^. Knockdown of *KDM5A* in these cells led to a significant reduction in the population of drug-tolerant cells^[62]^. Of note, KDM5A was found to exhibit an inverse expression relationship with BAK1 (BCL2-antagonist/killer 1), a protein that acts as a pro-apoptotic regulator. Hou *et al.*^[73]^ reported that deletion of *KDM5A* in a population of breast cancer cells with amplified *KDM5A*, resulted in up-regulation of *BAK1*, suggesting that KDM5A regulates the expression of this gene, amongst others.

In addition to this, KDM5A was also shown to mediate resistance to a second EGFR inhibitor, called gefitinib, in EGFR-mutant lung cancer cell lines^[77]^. In order to investigate whether KDM5A was actively involved in this phenotype, Gale *et al.*^[74]^ performed colony formation assays. Through these assays, the authors were able to show that fewer cells treated with a KDM5A inhibitor YUKA1, formed colonies during long-term treatment with gefitinib when compared to a population of control cells treated with DMSO (vehicle) and the same gefitinib long-term treatment^[74]^. Thus, the authors provided the first evidence that the demethylase activity of KDM5A is involved in gefitinib resistance in lung cancer cells^[74]^.

Two other KDMs, KDM1A and KDM5B were also found to play key roles in the development of hypoxia-induced resistance to gefitinib in patients with non-small cell lung cancer (NSCLC)^[67]^, albeit through a different mechanism than the one reported by Gale *et al.*^[74]^. Lu *et al.*^[67]^ were able to show that knockdown of KDM1A and KDM5B, prevented hypoxia-induced gefitinib resistance and also, inhibited epithelial-mesenchymal transition (EMT) that is critical for metastasis and drug resistance^[67,78]^. The results suggested that hypoxia is critical for the acquisition of resistance to EGFR TKIs, as a result of epigenetic change and mediation of EMT in NSCLC^[67]^.

In line with these results, KDM1A, was found to promote liver cancer cells’ resistance also to the TKI sorafenib^[72]^. In their publication, the authors reported an increased expression of *KDM1A* in sorafenib-resistance hepatocellular carcinoma cells. Furthermore, the inhibition of KDM1A using two potent KDM1A inhibitors, GSK2879552^[79]^ and pargyline^[80,81]^ was found to re-sensitize the same cells to the effect of sorafenib, partly through suppression of the Wnt/b-catenin signalling pathway and through reduction of the population of cancer stem cell-like cells^[72]^. Signalling mediated by the Wnt family of glycoproteins is one of the most important mechanisms that direct cell proliferation, polarity and determine cell fate during embryonic development and tissue homeostasis^[82]^. As a result of this, alterations in the Wnt pathway are often linked to cancer, amongst other diseases.

Other KDMs have also been implicated in the development of TKIs resistance, although their roles in this context need to be furtherly investigated. KDM5C was found to be a critical epigenetic modulator in the development of resistance to sunitinib in two different cancer cell lines of Lewis lung carcinoma and RCC^[75]^. Zimmermannova *et al.*^[76]^ have also reported the aberration of the *KDM6A* gene in imatinib-resistant cell lines of TEL-ABL-positive acute lymphoblastic leukaemia. In contrast with this finding, the aberration of this gene did not result in the expression of aberrant KDM6A protein^[76]^, so further research is required to fully determine the involvement of this demethylase in the development of TKI resistance in this cancer type.

Taken together this experimental evidence indicates that KDMs play key roles in the development of resistance to TKIs in several cancer types. Recent evidence also provides a suitable rationale to consider the use of new therapies that can be used to combat this phenomenon and prevent further development of cancer drug resistance. One approach could be to consider the use of specific KDM inhibitors to re-sensitize cells to tyrosine kinase target therapies. The data presented in this article also suggest that combination therapies that make use of TKIs and KDM inhibitors could possibly prevent, or reverse, the acquisition of resistance through epigenetic modulations and thus, could offer an attractive therapeutic strategy for certain cancers. In this context, it will be important to employ genetic and epigenetic stratification techniques to select patients that are more likely to benefit from the combination between TKIs and KDM inhibitors.

## Conclusion

To conclude, targeting KDMs is currently an active area of research in the development of new epigenetic drugs. Taking into account that many KDMs have been found to be amplified or overexpressed in a wide variety of human cancers and have been shown to play critical roles in mediating TKI resistance, these enzymes could be considered to be very attractive targets for the development of new therapeutic combinations.

## Declarations

### Copyright

© The Author(s) 2019.
